# Ferroptosis: a novel strategy to overcome chemoresistance in gynecological malignancies

**DOI:** 10.3389/fcell.2024.1417750

**Published:** 2024-07-09

**Authors:** Jing Xu, Bohao Zheng, Wei Wang, Shengtao Zhou

**Affiliations:** ^1^ Department of Obstetrics and Gynecology, Key Laboratory of Birth Defects and Related Diseases of Women and Children of MOE and State Key Laboratory of Biotherapy, West China Second Hospital, Sichuan University and Collaborative Innovation Center, Chengdu, Sichuan, China; ^2^ Department of Obstetrics and Gynecology, Chongqing Health Center for Women and Children, Women and Children’s Hospital of Chongqing Medical University, Chongqing, China; ^3^ Wuxi School of Medicine, Jiangnan University, Wuxi, Jiangsu, China; ^4^ Department of Pathology, West China Second Hospital, Sichuan University, Chengdu, Sichuan, China

**Keywords:** ferroptosis, gynecological malignancies, GPx4, iron metabolism, lipid peroxidation

## Abstract

Ferroptosis is an iron-dependent form of cell death, distinct from apoptosis, necrosis, and autophagy, and is characterized by altered iron homeostasis, reduced defense against oxidative stress, and increased lipid peroxidation. Extensive research has demonstrated that ferroptosis plays a crucial role in the treatment of gynecological malignancies, offering new strategies for cancer prevention and therapy. However, chemotherapy resistance poses an urgent challenge, significantly hindering therapeutic efficacy. Increasing evidence suggests that inducing ferroptosis can reverse tumor resistance to chemotherapy. This article reviews the mechanisms of ferroptosis and discusses its potential in reversing chemotherapy resistance in gynecological cancers. We summarized three critical pathways in regulating ferroptosis: the regulation of glutathione peroxidase 4 (GPX4), iron metabolism, and lipid peroxidation pathways, considering their prospects and challenges as strategies to reverse chemotherapy resistance. These studies provide a fresh perspective for future cancer treatment modalities.

## 1 Introduction

Gynecological malignancies, including cervical, ovarian, and endometrial cancers pose a significant threat to women’s health worldwide. Epidemiological data from 2020 revealed that approximately 1.34 million women were diagnosed with these malignancies, resulting in approximately 650,000 deaths. Notably, the mortality rate of endometrial cancer has steadily increased annually by approximately 1% (Siegel et al., 2023) Despite the twofold increase in the incidence of ovarian cancer in developed nations, the cumulative mortality risk remains similar between developed and developing regions (Zhang et al., 2019). Currently, the primary therapeutic modalities for these malignancies include surgery, chemotherapy, radiotherapy and targeted/immune therapies. Despite remarkable advances, modern medicine still faces the formidable challenge of drug resistance. This resistance not only reduces therapeutic efficacy and increases recurrence risks, but also imposes considerable financial burdens on healthcare systems (Guy et al., 2019; Vasan et al., 2019; Nie et al., 2022). Therefore, exploring the potential molecular mechanisms and therapeutic targets related to the treatment of chemoresistant gynecological malignancies is highly important.

Ferroptosis was initially characterized by Dixon et al., in 2012 as a distinct form of regulated cell death, intrinsically linked to iron accumulation (Tang et al., 2021). Unlike classic cell death pathways such as apoptosis, necrosis, and autophagy, the morphological features of ferroptosis include a significant reduction in cell volume, increased mitochondrial membrane density, disruption of membrane structures, decreased volume, and loss of mitochondrial cristae, while the nuclear structure typically remains unchanged (Trump et al., 1997; Saraste and Pulkki, 2000; Elmore, 2007; Mizushima and Yoshimori, 2007; Mizushima et al., 2011; Stockwell et al., 2017; Liu X. et al., 2022; Chen et al., 2022). These changes are induced by elevated levels of intracellular iron, which accelerates the generation of reactive oxygen species (ROS) through lipid peroxidation, leading to specific oxidative stress that damages mitochondria and lysosomes (Xie et al., 2016; Tang et al., 2019). In recent years, numerous studies have shown that ferroptosis plays a significant role in the pathogenesis and treatment of chemoresistance in gynecological malignancies. For example, ferroptosis inhibits the proliferation of ovarian cancer cells and their spread within the peritoneal cavity (Basuli et al., 2017) and can also reverse chemoresistance in ovarian cancer (Zhou et al., 2019). Thus, conducting in-depth research on ferroptosis may offer new opportunities for addressing chemoresistance in gynecological malignancies.

## 2 Mechanism of ferroptosis

**FIGURE 1:**
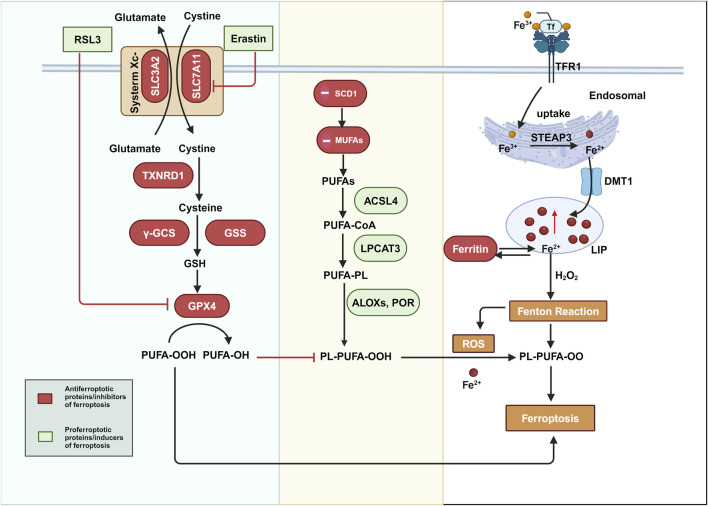
The three main regulatory pathways of ferroptosis: GPX4-regulated pathway, iron metabolism pathway, and lipid peroxidation pathway. Color coding and symbol usage: Red indicates proteins or inhibitors that suppress ferroptosis, green represents proteins or inducers that promote ferroptosis, and yellow is used specifically to denote biochemical processes such as the Fenton reaction and ferroptosis itself. Squares are used to mark chemical substances and biochemical processes, while ellipses are used to represent proteins and other biomolecules. Abbreviations: ROS, reactive oxygen species; GPX4, glutathione peroxidase 4; GSH, glutathione; xCT, the Xc-transport systemSLC7A11, solute carrier family 7 member 11; SLC3A2, solute carrier family 3 member 2; TXNRD1, thioredoxin reductase 1; γ-GCS, gamma-glutamylcysteine synthetase; GSS, glutathione synthetase; PUFA-OOH, polyunsaturated fatty acid hydroperoxides; PUFA-OH, polyunsaturated fatty acid alcohols; TFR1, transferrin receptor 1; DMT1, divalent metal transporter 1; PUFAs, polyunsaturated fatty acids; ACSL4, Acyl-CoA synthetase long-chain family member 4; LPCAT3, lysophosphatidylcholine acyltransferase 3; PUFA-CoA, polyunsaturated fatty acyl-CoA; PUFA-PL, polyunsaturated phospholipid; POR, cytochrome P450 oxidoreductase; PL-PUFA-OOH, phospholipid polyunsaturated fattyacid hydroperoxides; SCD1, stearoyl-CoA desaturase-1; MUFAs, monounsaturated fatty acids; ALOXs, arachidonate lipoxygenases; POR, Cytochrome P450 Oxidoreductase; LIP, labile iron pool.

Ferroptosis is a unique cell death mechanism. In-depth exploration of its molecular pathways not only aids in devising targeted interventions to induce cancer cell death but is also crucial for addressing chemotherapy resistance (Zhang et al., 2022). Since the seminal discovery of the glutathione peroxidase 4 (GPX4)-centered mechanism in 2014, the research momentum surrounding ferroptosis has intensified, with efforts increasingly focused on elucidating the complex pathways involved in this process (Yang et al., 2014). Based on the established GPX4-dependent pathway, emerging evidence has revealed several GPX4-independent pathways, and ultimately, three mainstream theories of ferroptosis mechanisms have been identified: the GPX4-regulated pathway, the iron metabolism pathway, and the lipid peroxidation pathway (Richardson and Ponka, 1997; Conrad and Pratt, 2019). These three pathways and the important molecules involved are summarized below and illustrated in Figure 1.

### 2.1 GPX4-regulated pathway

GPX4, a crucial selenoenzyme antioxidant, reduces phospholipid hydroperoxides (PLOOH) on the cell membrane, preventing oxidative damage caused by free radicals and maintaining the integrity of cell signaling and normal functions (Ursini et al., 1982). Additionally, some studies have shown that GPX4 works synergistically with GSH, a major intracellular antioxidant. Lipid-derived ROS are eliminated, thereby maintaining the cellular redox balance (Maiorino et al., 2018). The Xc-transport (xCT) system, composed of solute carrier family 7 member 11 (SLC7A11) and solute carrier family 3 member 2 (SLC3A2), functions as an intracellular cystine-glutamate antiporter and is essential for the synthesis of GSH (Bridges et al., 2012; Conrad and Sato, 2012; Koppula et al., 2018). This system plays a crucial role in maintaining the antioxidant status of cells, similar to GPX4. The system Xc-transports extracellular cystine into the cell while exporting intracellular glutamate, thereby maintaining amino acid balance (Lin et al., 2020).

Wang H. et al. (2021) demonstrated that downregulating the expression of GPX4 can induce ferroptosis in endometrial cancer cells and inhibit their growth. Similarly, Ye et al. (2021) showed that depleting glutathione (GSH) and inactivating GPX4 can also induce ferroptosis in cervical cancer cells, thereby enhancing the therapeutic effects against cervical cancer. Inside the cell, cystine is reduced to cysteine by thioredoxin reductase 1 (TXNRD1). This cysteine then combines with glutamate, catalyzed by γ-glutamylcysteine synthetase (γ-GCS), to form γ-glutamylcysteine (Andor et al., 2023; Griffith, 1999). Subsequently, γ-glutamylcysteine is converted into GSH in the presence of glycine, which is catalyzed by glutathione synthetase (GSS) (Zhu et al., 2023). GPX4 utilizes GSH to reduce polyunsaturated fatty acid hydroperoxides (PUFA-OOH) to polyunsaturated fatty acid alcohols (PUFA-OH), effectively preventing the accumulation of lipid peroxides (Ursini and Maiorino, 2020). When GPX4 activity is inhibited, such as by RSL3, PUFA-OOH accumulates within the cell, thereby inducing ferroptosis (Dai et al., 2020). Similarly, if the activity of SLC7A11 is inhibited by agents such as erastin, the intracellular transport of cystine is inhibited, leading to a reduction in GSH synthesis, indirectly inhibiting the activity of GPX4, and consequently leading to the accumulation of lipid peroxides and ferroptosis (Stockwell and Jiang, 2020). Therefore, modulating the GPX4 pathway to induce ferroptosis has contributed to the development of new anticancer strategies and enhanced the therapeutic efficacy against tumors.

### 2.2 Iron metabolism pathway

Ferritin, an iron storage protein, consists of heavy and light chain subunits. It can store a substantial amount of iron ions and catalyze the oxidation of Fe^2+^ at its iron oxidation center, preventing the formation of oxygen radicals through the Fenton reaction by free Fe^2+^, and inhibiting iron-induced oxidative stress (Theil, 1987; Arosio et al., 2009; Zhang J. et al., 2021). Iron is an essential micronutrient for cell growth, and its transport is critical for maintaining a balance between uptake and excretion. It is primarily transported into cells via transferrin and transferrin receptor 1 (TFR1), and is stored in ferritin (Zhou and Tan, 2017; Li et al., 2020). Under physiological conditions, iron ions (Fe^3+^), which enter the cell via TFR1, are reduced to ferrous ions (Fe^2+^) in the endoplasmic reticulum by STEAP3, a metalloreductase. While excessive intracellular Fe^2^⁺ can generate substantial amounts of hydroxyl radicals through the Fenton reaction, leading to oxidative stress, under physiological conditions, a balance between Fe^2^⁺ and Fe³⁺ is maintained to prevent such oxidative damage. Excess Fe^2+^ ions are transported by divalent metal transporter 1 (DMT1) within the cell and stored in ferritin to prevent excessive free iron from generating ROS (Bogdan et al., 2016). This internalization and storage process is vital for controlling ferroptosis (Stockwell et al., 2017).

The precise regulation of iron storage and excretion is primarily mediated by ferritin and the iron exporter solute carrier family 11a member 3 (SLC11A3) (Xie et al., 2016), which is involved in the export of iron ions, thus reducing the accumulation of iron within cells and effectively decreasing the production of ROS (McKie et al., 2000; Yang et al., 2002). Regulating iron metabolism has been proven to be an effective strategy for inducing ferroptosis in cancer cells (Chang et al., 2018). Using dysregulated iron metabolism in cancer cells, researchers have exploited the accumulated Fe^2+^ to increase the production of ROS through the Fenton reaction, thereby inducing ferroptosis in cancer cells (Winterbourn, 1995; Dixon and Stockwell, 2014). Research by Zhang YY. et al. (2021) has shown that quinone compounds, such as juglone, can disrupt iron homeostasis in Ishikawa endometrial cancer cells by modulating heme oxygenase and transferrin, leading to increased accumulation of Fe^2+^ and inducing ferroptosis, which inhibits the proliferation of endometrial cancer cells. Therefore, modulation of the iron metabolic pathway not only plays a crucial role in the occurrence of ferroptosis but also offers new targeted strategies for the treatment of gynecological malignancies.

### 2.3 Lipid peroxidation pathway

The peroxidation of polyunsaturated fatty acids (PUFAs) has been reported to produce PLOOH and subsequently generate 4-hydroxynonenal or malondialdehyde, which may lead to cell membrane damage, cellular dysfunction and ultimately ferroptosis (Conrad and Pratt, 2019; Chen et al., 2021; Liang et al., 2022). Acyl-CoA synthetase long-chain family member 4 (ACSL4) and lysophosphatidylcholine acyltransferase 3 (LPCAT3) play crucial roles in this process. They enhance the susceptibility of cell membranes to lipid peroxidation by promoting the acylation and re-esterification of PUFAs into phospholipids. ACSL4 and LPCAT3 catalyze the conversion of PUFAs into polyunsaturated fatty acyl-CoA (PUFA-CoA) and PUFA-CoA into polyunsaturated phospholipid (PUFA‐PL), respectively, thereby promoting the formation of lipid peroxides, which directly contribute to ferroptosis (Cui et al., 2023).

Additionally, the lipid peroxidation enzyme involved in lipid metabolism is vital for ferroptosis, and is primarily regulated by lipoxygenases (LOXs) and cytochrome P450 oxidoreductase (POR). LOXs, which are iron-containing enzymes, directly catalyze the transformation of both free and esterified PUFAs into lipid peroxides, thereby facilitating cell susceptibility to ferroptosis (Porter et al., 1995; Shah et al., 2018). Morever, POR, which acts downstream of cytochrome P450, accelerates the peroxidation of PUFA-containing lipids, particularly in the formation of phospholipid polyunsaturated fatty acid hydroperoxides (PL-PUFA-OOH), thereby promoting ferroptotic cell death (Ghosh et al., 1997; Zou et al., 2020).

Moreover, inhibiting stearoyl-CoA desaturase-1 (SCD1) shifts the cellular lipid composition toward increased saturated fatty acids and reduced monounsaturated fatty acids (MUFAs). This change decreases the availability of substrates for lipid peroxidation, potentially reducing cellular susceptibility to oxidative damage and inhibiting ferroptosis (Tesfay et al., 2019). Accordingly, precisely manipulating lipid metabolism pathways is a potential new approach for the treatment of diseases in which ferroptosis plays a key role.

## 3 Reversing chemoresistance in gynecological malignancies through ferroptosis

### 3.1 Ferroptosis in chemotherapy and chemoresistance of gynecological malignancies

In the treatment of gynecological malignancies, particularly ovarian, cervical, and endometrial cancers, chemotherapy remains one of the primary therapeutic approaches. Common chemotherapeutic drugs include platinum-based agents (such as cisplatin, carboplatin, and oxaliplatin) and taxanes. Platinum-based drugs and taxanes induce cell death through different mechanisms: the former through DNA damage and apoptosis (Kleih et al., 2019), and the latter by disrupting microtubule dynamics (Abal and Barasoain, 2003). Additionally, studies have shown that cisplatin and paclitaxel can enhance the efficacy of chemotherapy by inducing ferroptosis. Cisplatin acts by forming complexes with GSH, depleting intracellular GSH levels, and enhancing lipid peroxidation (Fu et al., 2023). Paclitaxel effectively induces ferroptosis by inhibiting the expression of SLC7A11, reducing GSH levels, and increasing oxidative stress and lipid peroxidation (Lv et al., 2017). These studies reveal the potential role of platinum-based drugs and taxanes in inducing ferroptosis, highlighting their importance in enhancing the efficacy of chemotherapy. Nonetheless, the presence of chemoresistance poses a significant challenge to the clinical application of these findings.

**FIGURE 2:**
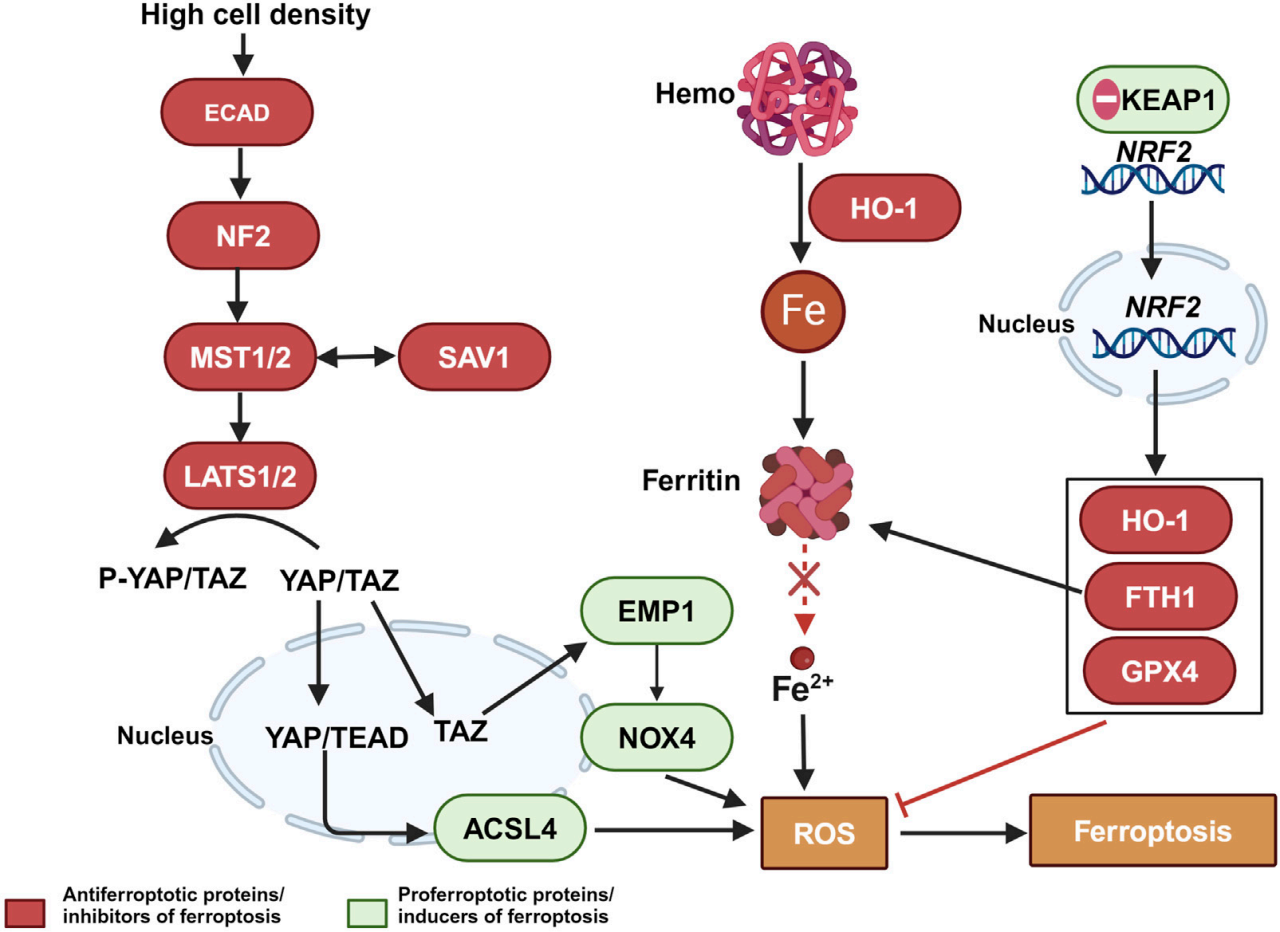
The relationship between ferroptosis escape and chemotherapy-resistant in tumor cells. Color coding and symbol usage: Red indicates proteins or inhibitors that suppress ferroptosis. Green represents proteins or inducers that promote ferroptosis. Yellow is used specifically to denote biochemical processes, including ROS. Squares are used to mark chemical substances and biochemical processes, while ellipses are used to represent proteins and other biomolecules. Abbreviations: MST1/2, Mammalian Ste20-like kinases 1 and 2; LATS1/2, Large tumor suppressor kinase 1 and 2; ECAD, E-Cadherin; YAP, Yes-associated protein; YAP/TAZ, Yes-associated protein and transcriptional coactivator with PDZ-binding motif; ACSL4, Acyl-CoA synthetase long-chain family member 4; EMP1, Epithelial Membrane Protein 1; NOX4, NADPH oxidase 4; ROS, reactive oxygen species; NRF2, Nuclear factor erythroid 2-related factor 2; KEAP1, Kelch-like ECH-associated protein 1; HO-1, Heme oxygenase-1; TFR1, transferrin receptor 1; Gpx4, glutathione peroxidase 4.

Furthermore, dysregulated iron metabolism is a key characteristic of malignant tumors. The increased demand for iron in tumor cells leads to elevated levels of TFR1 and ferritin light chain (FTL) (Ludwig et al., 2015; Adachi et al., 2019). Abnormal iron metabolism plays a crucial role in tumor development, survival, proliferation, and metastasis, making it an important focus in cancer treatment (Shen YLX. et al., 2018; Guan and Zhu, 2023). Chemoresistant cancer cells often exhibit abnormally high intracellular iron levels, which may be key factors in chemotherapy resistance and poor prognosis, such as breast cancer (Chekhun et al., 2013; Conti et al., 2016; Kazan et al., 2017; Shen et al., 2018b), esophageal cancer (Wang et al., 2022) pancreatic ductal carcinoma (Song et al., 2021). Specifically, increased expression of the ferritin light chain may help cancer cells store more iron, thereby reducing ferroptosis. This mechanism is associated with chemoresistance in cancer cells (Chekhun et al., 2013). Additionally, defense mechanisms in chemoresistant cancer cells can further inhibit ferroptosis, enhancing resistance to chemotherapeutic drugs. These mechanisms include the activation of the Hippo pathway, regulated by the kinases MST1/2 and LATS1/2, which are controlled by the cell adhesion molecule E-cadherin under conditions of high cell density. This leads to a reduction in the nuclear presence of the transcription coactivators YAP/TAZ and a decrease in the expression of ferroptosis-promoting proteins such as ACSL4, epithelial membrane protein 1 (EMP1), and NADPH oxidase 4 (NOX4), thereby inhibiting ferroptosis (Yang et al., 2019; He et al., 2022; Cui et al., 2023). Additionally, the ferroptosis regulator nuclear factor erythroid 2-related factor 2 (NRF2), under the regulation of the antioxidant response protein Kelch-like ECH-associated protein 1 (KEAP1), translocates to the nucleus, resulting in increased expression of anti-ferroptosis proteins such as heme oxygenase-1 (HO-1), FTH1, and GPX4, thus resisting ferroptosis (Song et al., 2024). These regulatory factors play a crucial role in combating chemotherapy-induced cell death, underscoring the potential of modulating ferroptosis-related molecular pathways to reverse chemoresistance (as shown in Figure 2).

Given these findings, an in-depth exploration of the therapeutic potential of targeting specific ferroptotic pathways has emerged as a new focus in the treatment of gynecological malignancies. By focusing on the GPX4 regulatory pathway, iron metabolism, and lipid peroxidation, we propose a novel strategy to overcome chemoresistance and enhance the efficacy of gynecological cancer therapies.

### 3.2 Targeting the Gpx4-regulatory pathway

Research has shown that GPX4 dysfunction is associated with tumor cell resistance (Hangauer et al., 2017). In gynecological malignancies, cell lines resistant to chemotherapeutic drugs such as cisplatin, carboplatin, and paclitaxel have been found to increase GPX4 expression and activity by modulating the expression of ferroptosis-related genes, thereby resisting ferroptosis induction. For instance, in the ovarian cancer-resistant cell line CAOV2R, downregulation of the transcriptional coactivator with PDZ-binding motif (TAZ) leads to increased expression and activity of GPX4, thereby enhancing cells resistance to ferroptosis and contributing to carboplatin resistance. Conversely, overexpression of TAZ can decrease GPX4 activity, reverse resistance, and increase sensitivity to carboplatin (Yang et al., 2020). In the HEC-1A/DDP and Ishikawa/DDP cell lines, overexpression of protein arginine methyltransferase (PRMT3) decreased the m6A modification of GPX4 mRNA, which not only reduced the expression of the GPX4 protein but also impaired its function, thereby inhibiting ferroptosis and leading to cisplatin resistance. PRMT3 inhibitors, such as SGC707, can restore the normal m6A modification of GPX4 mRNA, reversing cisplatin resistance in endometrial cancer cells (Wang Y. et al., 2023). Similarly, increased expression of SCD1 and fatty acid desaturase 2 (FADS2) leads to enhanced GPX4 activity, resulting in resistance to cisplatin in the ovarian cancer cell line PEO4. The use of inhibitors of SCD1 and FADS2 can reduce GPX4 activity and enhance the sensitivity of ovarian cancer cells to cisplatin (Xuan et al., 2022). In the ES-A cell line, increased expression of xCT leads to elevated levels of GSH and GPX4, inhibiting ferroptosis and resulting in resistance to paclitaxel. However, combined treatment with paclitaxel (PTX) and sulfasalazine (SAS) can reduce GPX4 levels, increase intracellular iron content and ROS accumulation, enhance sensitivity to paclitaxel, and reverse resistance (Wang Y. et al., 2021). These findings highlight the central role of GPX4 in chemoresistance and suggest the potential for targeting GPX4 to reverse resistance.

Additionally, GPX4 activity is closely related to the increased expression of SLC7A11, a component of the cystine/glutamate antiporter that influences intracellular cystine levels and thereby affects GSH synthesis. Changes in the levels of GSH, the essential coenzyme of GPX4, directly affect the enzyme’s ability to reduce lipid hydroperoxides, thus influencing its antioxidant capacity and the regulation of ferroptosis (Endale and Mengstie, 2023). Therefore, the overexpression of SLC7A11 in ovarian cancer cells enhances GPX4 activity by increasing GSH levels, which assists cells in resisting ferroptosis and promotes cancer cell proliferation, invasion, and chemoresistance (Fantone et al., 2024). Qin et al. (2023) also reported that SLC7A11 protein levels were significantly greater in cisplatin-resistant A2780/DDP and SKOV3/DDP ovarian cancer cell lines than in their cisplatin-sensitive counterparts. Silencing circSnx12 increased the expression of miR-194-5p, which can reduce SLC7A11 expression, thereby increasing cisplatin sensitivity and reversing resistance.

Moreover, chemoresistant cells evade ferroptosis by enhancing antioxidant defense mechanisms (Viswanathan et al., 2017). GPX4, a key antioxidant enzyme, utilizes the reducing power provided by nicotinamide adenine dinucleotide phosphate (NADPH) to reduce lipid peroxides, thereby resisting ferroptosis (Ye et al., 2024). Lowering NADPH levels weakens GPX4’s antioxidant capacity, increasing the sensitivity of cells to GPX4 inhibitors and suggesting new possibilities for inducing ferroptosis. In a study involving 192 ovarian cancer patients, high levels of GPX4 were associated with resistance to platinum-based drugs, and siRNA-mediated knockdown of GPX4 reduced the resistance of ovarian cancer cells to these drugs (Wu et al., 2022). Based on these findings, the research and development of GPX4 inhibitors have become promising. For example, the GPX4 inhibitor RSL3 effectively inhibits the proliferation of the medroxyprogesterone acetate (MPA)-resistant endometrial cancer cell line ECC-1 by enhancing oxidative stress and inducing ferroptosis, thereby reversing resistance (Murakami et al., 2023). Luo et al. (2022) designed a GPX4 degrader, dGPX4, that degrades GPX4 in tumor cells via the proteasome pathway, achieving an efficiency five times greater than that of inducing ferroptosis with ML162, a GPX4 inhibitor. Additionally, they developed biodegradable lipid nanoparticles (dGPX4@401-TK-12) to deliver dGPX4 intracellularly, targeting the cancer cell microenvironment to induce selective ferroptosis. When administered intravenously, these nanoparticles effectively inhibited tumor growth without significant side effects.

### 3.3 Targeting the iron metabolism pathway

Ferritinophagy is a selective autophagy process mediated by coactivator 4 (NCOA4), which releases stored iron by degrading ferritin in lysosomes, helping chemoresistant cancer cells maintain high iron levels. Therefore, chemoresistant cells can adapt to oxidative stress induced by chemotherapeutic drugs by enhancing ferritinophagy, thereby increasing their tolerance to these drugs (Terman and Kurz, 2013; Zhou et al., 2020; Yu et al., 2022). However, when ferritinophagy is excessively activated, it can lead to an imbalance in the iron pool, triggering the Fenton reaction and ultimately inducing ferroptosis (Masaldan et al., 2018; Yu et al., 2022). Research by Qiu et al. (2021) confirmed this finding, showing that downregulation of NCOA4 is associated with drug resistance, while overexpression of NCOA4 promotes ferritin degradation, and triggers ferroptosis, thereby enhancing sensitivity to chemotherapeutic drugs, and reversing resistance. Furthermore, dihydroartemisinin (DHA) induces NCOA4-mediated ferritinophagy in cervical cancer, leading to increased labile iron pool (LIP), enhanced Fenton reaction, and excessive ROS production, triggering ferroptosis, and sensitizing cervical cancer cells to doxorubicin (Shi et al., 2023).

Chemoresistance is also closely related to changes in iron metabolism. In ovarian cancer cells, the expression of TFR1, DMT1, and hepcidin (HAMP) is increased, while the expression of FPN is decreased. This leads to elevated intracellular iron concentrations and increased levels of FTL, contributing to resistance to chemotherapeutic drugs (Arezes and Nemeth, 2015; Xue et al., 2016; Basuli et al., 2017; Wang et al., 2019). The iron chelator desferrioxamine (DFO) can deplete iron required by tumor cells, reduce the activation of tumor cell enzymes, exhibit anticancer activity (Han et al., 2019; Recalcati et al., 2019). Existing literature suggested that DFO can restore the drug sensitivity in cisplatin-resistant cells by altering mitochondrial iron metabolism. Thus, the reduced cisplatin dose can diminish the side effects of the chemotherapy (Liu WJ. et al., 2022). Wang et al. (2019) confirmed that DFO effectively inhibits the proliferation of SKOV-3 and OVCAR-3 ovarian cancer cells, enhances the efficacy of cisplatin treatment, and reverses cisplatin resistance in ovarian cancer.

### 3.4 Targeting the lipid peroxidation pathway

SCD1 is a key enzyme in the lipid oxidation pathway, and is primarily responsible for catalyzing the conversion of saturated fatty acids to monounsaturated fatty acids (Bednarski et al., 2016; Tesfay et al., 2019). Studies have shown that SCD1 can promote chemoresistance in tumors by inhibiting ferroptosis (Zhang H. et al., 2021; Luis et al., 2021). In study on gynecological malignancies, Xuan et al. (2022) reported that SCD1 is abnormally upregulated in ascites-derived ovarian cancer (OvCa) cells, and is closely associated with tumor invasiveness and chemoresistance. Using the SCD1 inhibitor CAY10566 or silencing SCD1 via CRISPR/Cas9 technology can delay tumor growth, reduce the formation of cancer stem cells, and decrease resistance to platinum-based drugs. Additionally, another study demonstrated that in chemoresistant ovarian cancer cells (SKOV3-CIS and A2780-CIS), inhibiting SCD1 expression by activating the AMPKα signaling pathway can induce ferroptosis, enhance the sensitivity of ovarian cancer cells to platinum-based drugs, and reverse chemoresistance (Tang et al., 2022).

In addition to SCD1, LOXs also play important roles in ferroptosis. LOXs mediate ferroptosis through the direct oxygenation of polyunsaturated fatty acids (Kuhn et al., 2015). For example, downregulation of arachidonate 15-lipoxygenase (ALOX15) has been reported to enhance sensitivity to cisplatin and paclitaxel in breast cancer (Li et al., 2016). However, in endometrial cancer cell lines (AN3CA and Ishikawa), downregulation of aarF domain containing kinase 3 (ADCK3) reduces the transcription of ALOX15, affecting lipid peroxidation and inhibiting ferroptosis, thereby leading to MPA resistance. Silencing or knocking out ADCK3 via siRNA or CRISPR/Cas9 technology can enhance lipid peroxidation, promote ferroptosis, and reverse MPA resistance (Zhang et al., 2023).

Another key factor is the iron-sulfur protein ferredoxin 1 (FDX1), which plays a crucial role in the biosynthesis of iron-sulfur clusters and steroidogenesis (Grinberg et al., 2000; Sheftel et al., 2010). FDX1 is important for maintaining cellular iron homeostasis. Depletion of FDX1 not only leads to iron homeostasis disruption but also causes mitochondrial iron overload, both of which are key drivers of ferroptosis (Shi et al., 2012). Studies have shown that FDX1 is upregulated in platinum-resistant ovarian cancer cell lines (A2780/DDP and SKOV3/DDP), inhibiting ferroptosis and resulting in cisplatin resistance. Silencing the FDX1 gene via siRNA can enhance lipid peroxidation, promote ferroptosis, and promote cisplatin resistance (Takahashi et al., 2023).

In summary, the GPX4 pathway, iron metabolism pathway, and lipid peroxidation pathway play crucial roles in reversing chemotherapy resistance. By precisely regulating key genes within these pathways, we can effectively restore or enhance the induction of ferroptosis, thereby reversing the resistance of tumor cells to chemotherapeutic drugs. Table 1 lists the gene changes induced by chemotherapy drugs, providing potential targets for focusing on these pathways.

**TABLE 1:** Key ferroptosis related gene and protein targets in chemo-resistant gynecological cancer cell line.

Drugs	Tumor	Cell line	Gene	Expression	Target pathway	Resistance mechanism	Reversal method	References
Carboplatin	Ovarian cancer	CAOV2R	TAZ	↓	GPX4-regulated pathway	siRNA-mediated knockdown of TAZ reduces ANGPTL4, decreases NOX2 activation, increases GPX4 expression, leading to ferroptosis resistance	Overexpressing TAZ	Hangauer et al. (2017)
Cisplatin	Ovarian Cancer	A2780/DDP SKOV3/DDP	FDX1	↑	Lipid metabolism pathway	FDX1 downregulation affects mitochondrial membrane potential and lipid peroxidation, inhibiting ferroptosis	siRNA-mediated FDX1	Shi et al. (2012)
		PEO4	SCD1 FADS2	↑ ↑	GPX4-regulated pathway	Overexpression of SCD1/FADS2 increases GPX4 expression, inhibiting ferroptosis	Inhibition of SCD1 and FADS2	Wang et al. (2023a)
	Endometrial cancer	HEC-1A/DDP Ishikawa/DDP	PRMT3	↑	GPX4-regulated pathway	PRMT3 overexpression decreases m6A modification of GPX4 mRNA, and inhibits ferroptosis	SGC707 (PRMT3 inhibitor) sh-PRMT3	Yang et al. (2020)
Paclitaxel	Ovarian cancer	ES-2	xCT	↑	GPX4-regulated pathway	xCT overexpression elevates GSH and GPX4, inhibiting ferroptosis	Combined treatment with PTX and SAS	Xuan et al. (2022)
MPA	Endometrial cancer	AN3CA Ishikawa	ADCK3	↓	Lipid metabolism pathway	ADCK3 downregulation affects lipid peroxidation, inhibiting ferroptosis	Silencing or knocking out ADCK3 using siRNA or CRISPR/Cas9	Li et al. (2016)

## 4 Prospects of ferroptosis in the treatment of gynecological malignancies

### 4.1 Ferroptosis and traditional treatment

In the treatment of gynecological malignancies, the combined use of ferroptosis inducers has shown significant potential with conventional treatment modalities such as chemotherapy, targeted therapy and radiotherapy. In recent years, ferroptosis inducers have demonstrated significant efficacy in overcoming chemoresistance. For instance, the use of erastin can reduce the efflux of the multidrug resistance protein ATP-binding cassette subfamily B member 1 (ABCB1), leading to increased accumulation of chemotherapeutic drugs within tumor cells, thereby reversing resistance to docetaxel and platinum-based drugs (Zhou et al., 2019). This finding indicates that the combined use of ferroptosis inducers and chemotherapy drugs can enhance chemotherapy efficacy by modulating the ferroptosis pathway. Ferroptosis inducers activate ferroptosis by promoting lipid peroxidation and increasing intracellular iron ion concentrations, while chemotherapy drugs indirectly support this process by increasing the production of ROS (Wang YWX. et al., 2023). This synergistic effect not only enhances the sensitivity of tumor cells to chemotherapy drugs but also helps overcome chemoresistance, offering new strategies for the treatment of gynecological malignancies. Additionally, tumor heterogeneity particularly complicates the management of treatment resistance, tumor progression, and recurrence (Fidler, 1978; Hallou et al., 2017). Li et al. (2021) revealed that in multiple ovarian cancer cell lines, the majority (10 out of 11) exhibited high expression of the autophagy marker MAP1LC3B-II, which was positively correlated with sensitivity to ferroptosis induced by erastin or RSL3. This discovery suggests that effective personalized treatment strategies can be developed based on the differential sensitivity of various tumor subtypes to ferroptosis inducers.

In the field of targeted therapy, olaparib, a well-known PARP inhibitor, is primarily used for treating patients with BRCA-mutated ovarian cancer. However, its efficacy is often limited in patients with wild-type BRCA1/2. Studies have shown that enhancing ferroptosis through the use of ferroptosis inducers (FINs) can synergize with PARP inhibitors, increasing the sensitivity of non-BRCA-mutated ovarian cancer cells and their xenografts (Hong et al., 2021). This strategy offers a new therapeutic direction for the use of PARP inhibitors in the treatment of ovarian cancer patients with functional BRCA. In addition to their applications in chemoresistance and targeted therapy, ferroptosis inducers also show potential in radiotherapy. Radiotherapy kills cancer cells by inducing oxidative stress and lipid peroxidation. However, it also induces the expression of SLC7A11 and GPX4, proteins that help cells resist ferroptosis in cancer cells, thereby reducing treatment efficacy (Lang et al., 2019). By depleting or inhibiting SLC7A11 or GPX4, such as by using erastin (an SLC7A11 inhibitor) or RSL3 (a GPX4 inhibitor), radiotherapy-induced ferroptosis can be enhanced, significantly increasing radiotherapy sensitivity (Xie et al., 2011; Pan et al., 2019; Lei et al., 2020). This phenomenon is also observed in ovarian cancer, where FINs enhance the effects of radiotherapy by inhibiting SLC7A11-mediated cystine uptake or GPX4 activity. In patient-derived ovarian cancer tumor organoids, the combination of FINs and radiotherapy significantly enhanced cell death induction and reduced cell viability (Lei et al., 2021). Similarly, Lang et al. (2019) pretreated ID8 ovarian cancer cells with ferroptosis inducers (such as erastin and FINs) before radiation exposure, and found that this pretreatment enhanced the sensitivity of ovarian cancer cells to radiotherapy.

In summary, these strategies not only hold promise for enhancing the efficacy of existing therapies, but also pave the way for new avenues in clinical research on the treatment of gynecological malignancies through ferroptosis.

### 4.2 Ferroptosis and emerging technologies

Facing therapeutic challenges, researchers have proposed a series of innovative treatment strategies, that showcasing the potential of ferroptosis in transforming the field of cancer therapy. In this domain, using radiomic features extracted from dynamic contrast-enhanced magnetic resonance imaging (DCE-MRI), Su et al. (2023) highlighted the significance of ferroptosis as a therapeutic target in highly heterogeneous tumors. These techniques enable researchers to more precisely identify and target tumor subtypes that may be more sensitive to ferroptosis inducers. Additionally, Cao et al. (2024) identified methotrexate sodium as an effective inhibitor of GPX4 through fluorescence polarization, a method that surpasses traditional cell phenotype screening, providing a more precise approach for selecting potent ferroptosis inhibitors. Concurrently, Chen et al. (2020) utilized the IVIS bioluminescence imaging system to track biomarker changes during the ferroptosis process, thereby validating the efficacy of therapeutic strategies in real time.

With technological advancements, the application of nanotechnology in ferroptosis research has increased. Nanoparticles have served as primary carriers for ferroptosis inducers in several studies. Nanoiron (Nano-Fe) can release iron ions within tumor cells, triggering the Fenton reaction and inducing ferroptosis. Due to the high demand for iron by tumor cells, these nanoparticles are preferentially taken up in large amounts by tumor cells, while uptake by normal tissues remains minimal, enhancing the targeting of the therapy. Moreover, the release of iron ions can be monitored via magnetic resonance imaging (MRI), offering the possibility of real-time monitoring of the treatment, which further improves the safety and efficacy of the therapy (Shen et al., 2017; Shen et al., 2018c; Hassannia et al., 2019; Liu et al., 2019; Sang et al., 2019). For instance, Guan et al. (2020) designed SRF@MPDA-SPIO nanoparticles, a technology that loads sorafenib onto ultrasmall superparamagnetic iron oxide (SPIO) nanoparticles. These nanoparticles not only leverage their targeting ability to deliver sorafenib directly to tumor cells but also simultaneously release iron ions, which can be monitored via MRI, thereby enhancing the efficacy and safety of the treatment. Similarly, Fan et al. (2023) designed magnetic iron oxide nanoparticles with AND logic gate functionality. This design exploits the unique biochemical signals present in the tumor microenvironment to activate the nanoparticles, significantly enhancing the targeting and efficacy of ferroptosis therapy. Guided by MRI, these nanoparticles can precisely release iron ions within tumor cells, inducing ferroptosis without affecting surrounding normal cells. These innovative methods and technologies bring new hope for the treatment of cancer with dysregulated iron metabolism including gynecological malignancies. Future research will continue to explore how to maximize the efficacy of ferroptosis inducers and translate these findings into clinical practice.

## 5 Conclusion

The role of ferroptosis in the treatment of gynecological malignancies, particularly in combating chemoresistance, has garnered widespread attention. Utilizing the three key pathways of ferroptosis—GPX4 regulation, iron metabolism, and lipid peroxidation-to develop new therapeutic strategies has shown significant potential in overcoming chemoresistance. Research by Wang et al. (2023) further underscores the effectiveness of ferroptosis inducers in overcoming resistance to chemotherapeutic drugs. However, the clinical translation of ferroptosis-based therapies still faces numerous challenges, and the underlying mechanisms are highly complex. In addition to the aforementioned three key pathways, many other pathways remain to be explored. So far, no ongoing clinical trials targeting ferroptosis in gynecological malignancies have been documented. Therefore, more in-depth research and attempt for ferroptosis-targeted clinical trials are in dire need to identify more effective therapeutic approaches for the treatment of gynecological malignancies.
